# Supplementary material for: Dan forms condensates in neuroblasts and regulates nuclear architecture and progenitor competence in vivo
Source: Nat Commun. 2024 Jun 14;15:5097. doi: 10.1038/s41467-024-49326-6 (PMC11178893; doi:10.1038/s41467-024-49326-6)
Supplement: Supplementary file 1 — Supplementary Information [file 41467_2024_49326_MOESM1_ESM.pdf]

Supplemental information for

Dan forms condensates in neuroblasts and regulates nuclear architecture and progenitor competence *in vivo*

Gillie Benchorin, Richard Jangwon Cho, Maggie Jiaqi Li, Natalia Molotkova and Minoree Kohwi

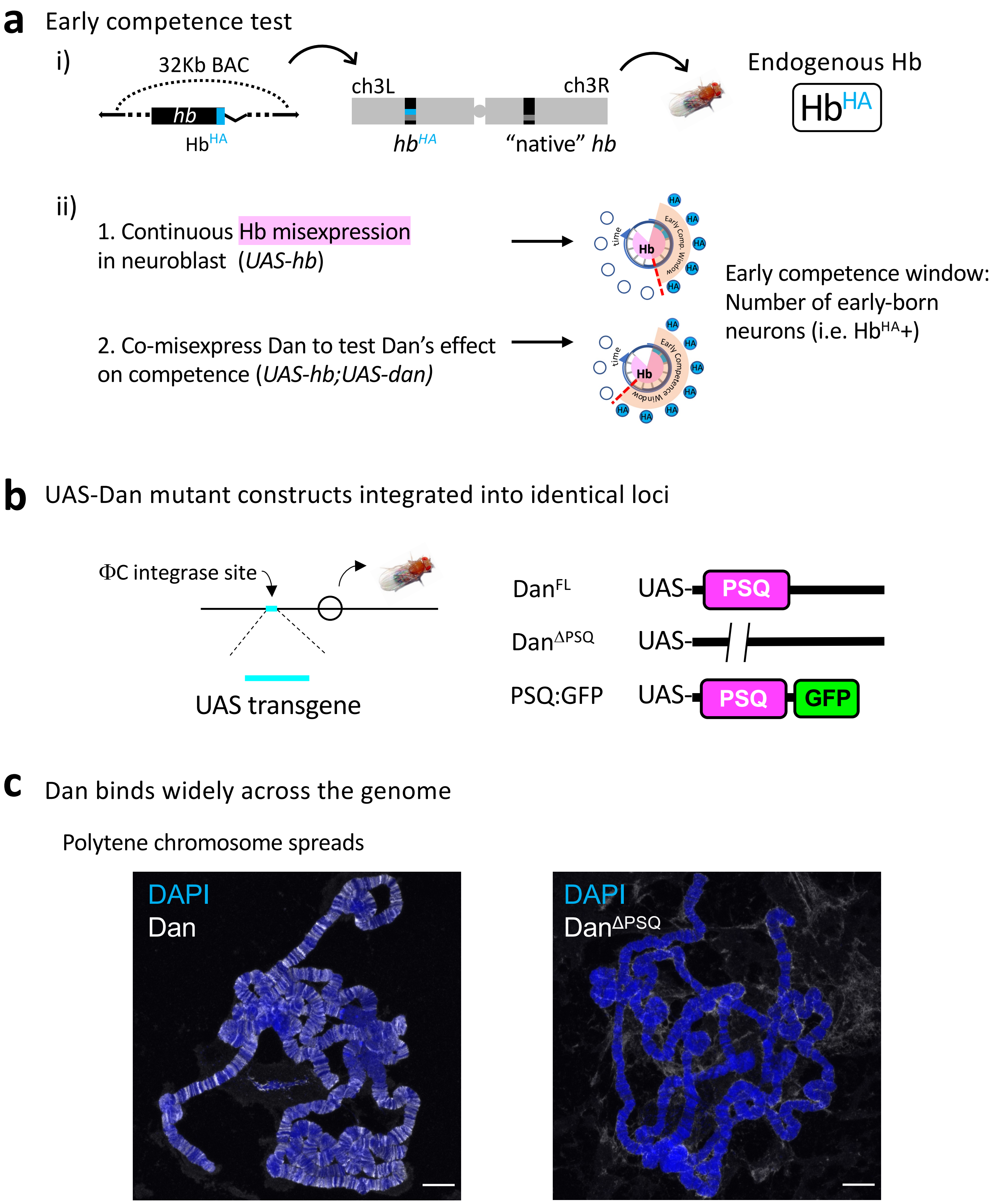
a
Early competence test
i)
32Kb BAC
hb
HbHA
ch3L
ch3R
hbHA
"native" hb
Endogenous Hb
HbHA
ii)
1. Continuous Hb misexpression
in neuroblast (UAS-hb)
2. Co-misexpress Dan to test Dan's effect
on competence (UAS-hb;UAS-dan)
time
Hb
Early Comp. Window
HA
Early competence window:
Number of early-born
neurons (i.e. HbHA+)
b
UAS-Dan mutant constructs integrated into identical loci
ΦC integrase site
UAS transgene
DanFL
UAS-
PSQ
DanΔPSQ
UAS-
PSQ:GFP
UAS-
PSQ
GFP
c
Dan binds widely across the genome
Polytene chromosome spreads
DAPI
Dan
DAPI
DanΔPSQ

Supplemental Figure 1 (Supplement to Figure 1)

ai, Schematic diagram of early competence test. Endogenous Hb is expressed from a bacterial artificial chromosome (BAC) modified to fuse at HA epitope to Hb that was integrated into chromosome 3L (ch3L). HA thus acts as a proxy for endogenous Hb expression and can be distinguished from Hb misexpressed via the Gal4/UAS system. See *Lucas* et al, 2021[7]. **ii,** To test Dan's effects on competence, Dan and Hb are co-misexpressed in the neuroblast, and HA^{+} neurons are quantified.

b, UAS-Dan mutant constructs are integrated into the same genomic loci using the phiC site-specific integration.

c, Squashed salivary glands immunostained for myc tag fused to Dan^{FL} and $Dan^{\Delta PSQ}$ show that Dan requires the Psq DNA-binding domain to bind the genome.

Scale bars, 10µm.

Drosophila image by André Karwath (Aka) shared in Wikipedia under a CC BY-SA 2.5 Licence (https://creativecommons.org/licenses/by-sa/2.5/)

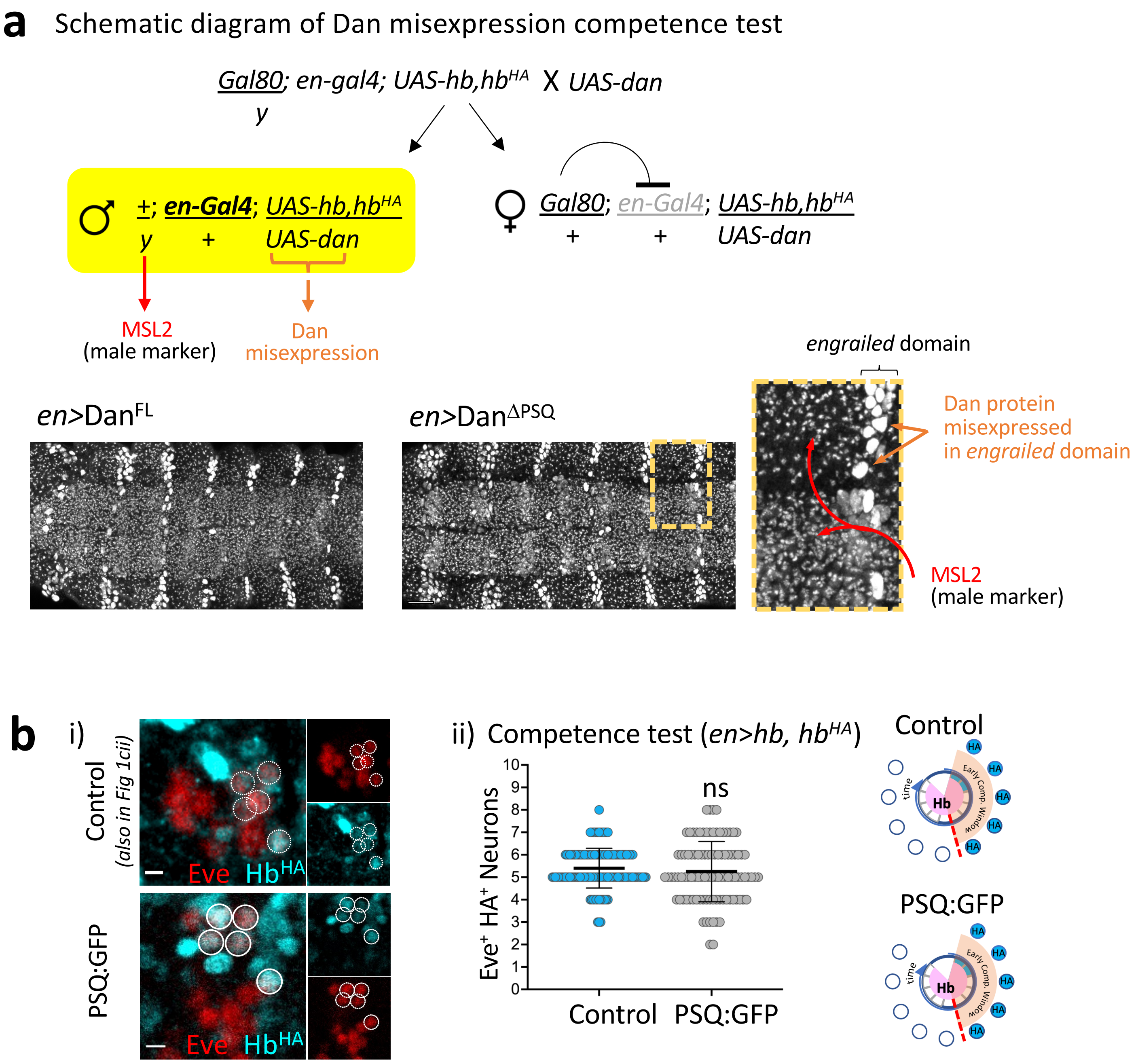
a
Schematic diagram of Dan misexpression competence test
Gal80; en-gal4; UAS-hb,hbHA X UAS-dan
y
♂ +; en-Gal4; UAS-hb,hbHA
y + UAS-dan
♀ Gal80; en-Gal4; UAS-hb,hbHA
+ + UAS-dan
MSL2
(male marker)
Dan
misexpression
engrailed domain
en>DanFL
en>DanΔPSQ
Dan protein
misexpressed
in engrailed domain
MSL2
(male marker)
b
i)
Control
(also in Fig 1cii)
Eve HbHA
PSQ:GFP
Eve HbHA
ii) Competence test (en>hb, hbHA)
Eve+ HA+ Neurons
ns
Control
PSQ:GFP
Control
PSQ:GFP
time
Hb
Early Comp. Window
HA

Supplemental Figure 2 (Supplement to Figure 1)

a, Schematic diagram of Dan misexpression competence test. Msl2 marker staining identifies male embryos that do not carry the *Gal80* allele, and therefore have an active *en-Gal4* driver for Hb and Dan misexpression. Z-projections of MSL2 and *en*>Dan expression in the ventral nerve cord. MSL2 appears as small nuclear puncta, while Dan can be seen expressed in engrailed stripes and throughout the nerve cord, thus confirming Dan misexpression. Scale bars, 20μm.

b, NB7-1 lineage early competence assay. **i** representative images showing NB7-1 lineage misexpressing Hb only (control) or co-misexpressing Hb and PSQ:GFP. Scale bars, 3μm. **ii,** Graph shows quantified data of *i*; Control n=142 NB7-1 lineages from 8 embryos, PSQ n=100 from 5 embryos. Error bars represent mean ± SD (two-tailed unpaired *t*-test, ns, not significant, *p*=0. 2915). Source data are provided as a Source Data file.

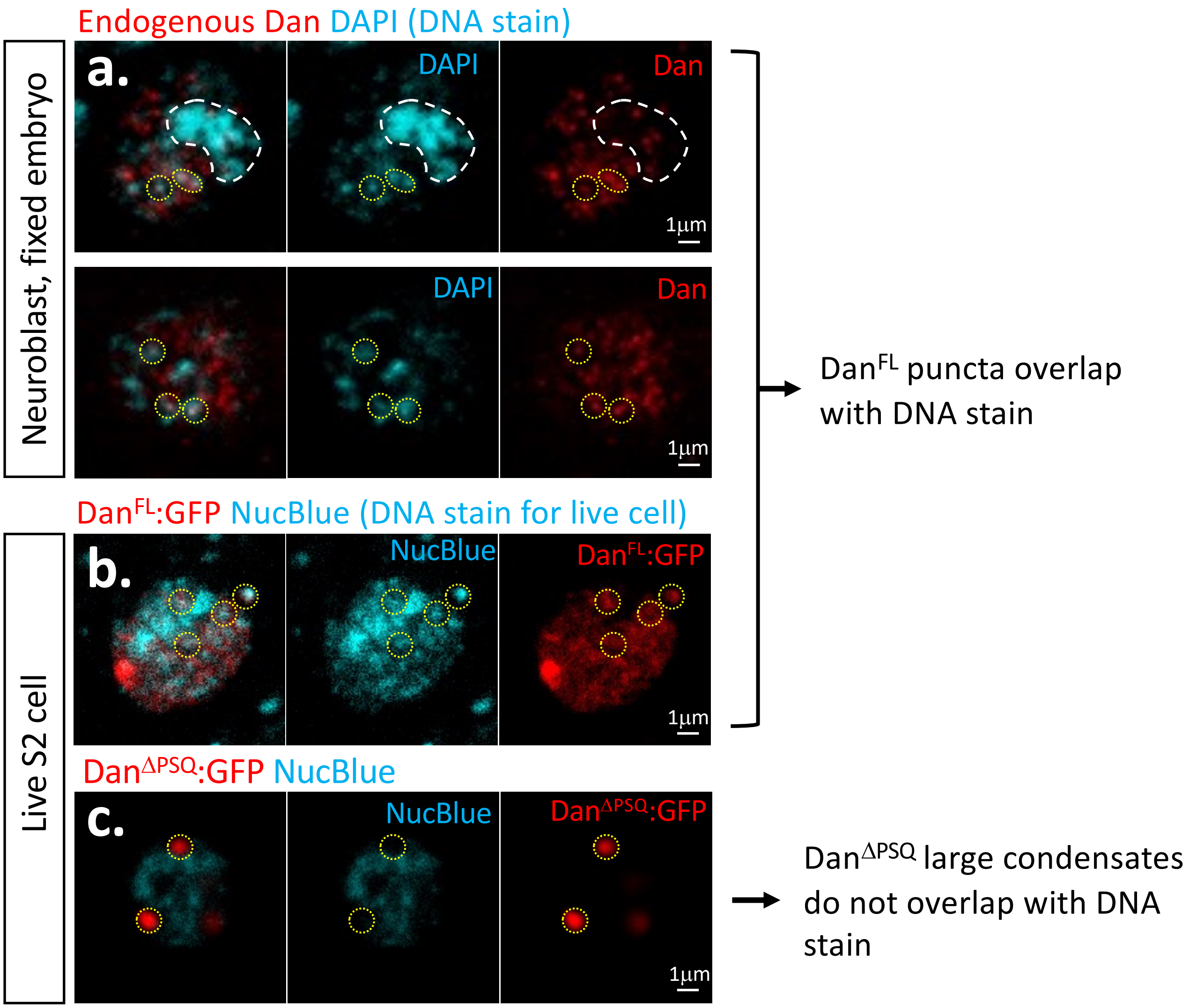
Endogenous Dan DAPI (DNA stain)
Neuroblast, fixed embryo
a.
DAPI
Dan
1μm
DAPI
Dan
1μm
Dan^FL puncta overlap with DNA stain
Dan^FL:GFP NucBlue (DNA stain for live cell)
Live S2 cell
b.
NucBlue
Dan^FL:GFP
1μm
Dan^ΔPSQ:GFP NucBlue
c.
NucBlue
Dan^ΔPSQ:GFP
1μm
Dan^ΔPSQ large condensates do not overlap with DNA stain

Supplemental Figure 3 (Supplement to Figure 2)

Dan protein distribution relative to chromatin.

a, two examples of neuroblasts from fixed embryos showing endogenous Dan (red) and the DNA stain, DAPI (cyan). Note Dan puncta overlap with DAPI (yellow circles).

b, Full length Dan (Dan^{FL}:GFP, red) expressed in S2 cells, imaged live. Similar to endogenous Dan in fixed embryos, Dan^{FL}:GFP puncta overlap with DNA stain (NucBlue, cyan).

c, $Dan^{\Delta PSQ}$:GFP (red) large condensates avoid DNA stains. Scale bars, 1μm.

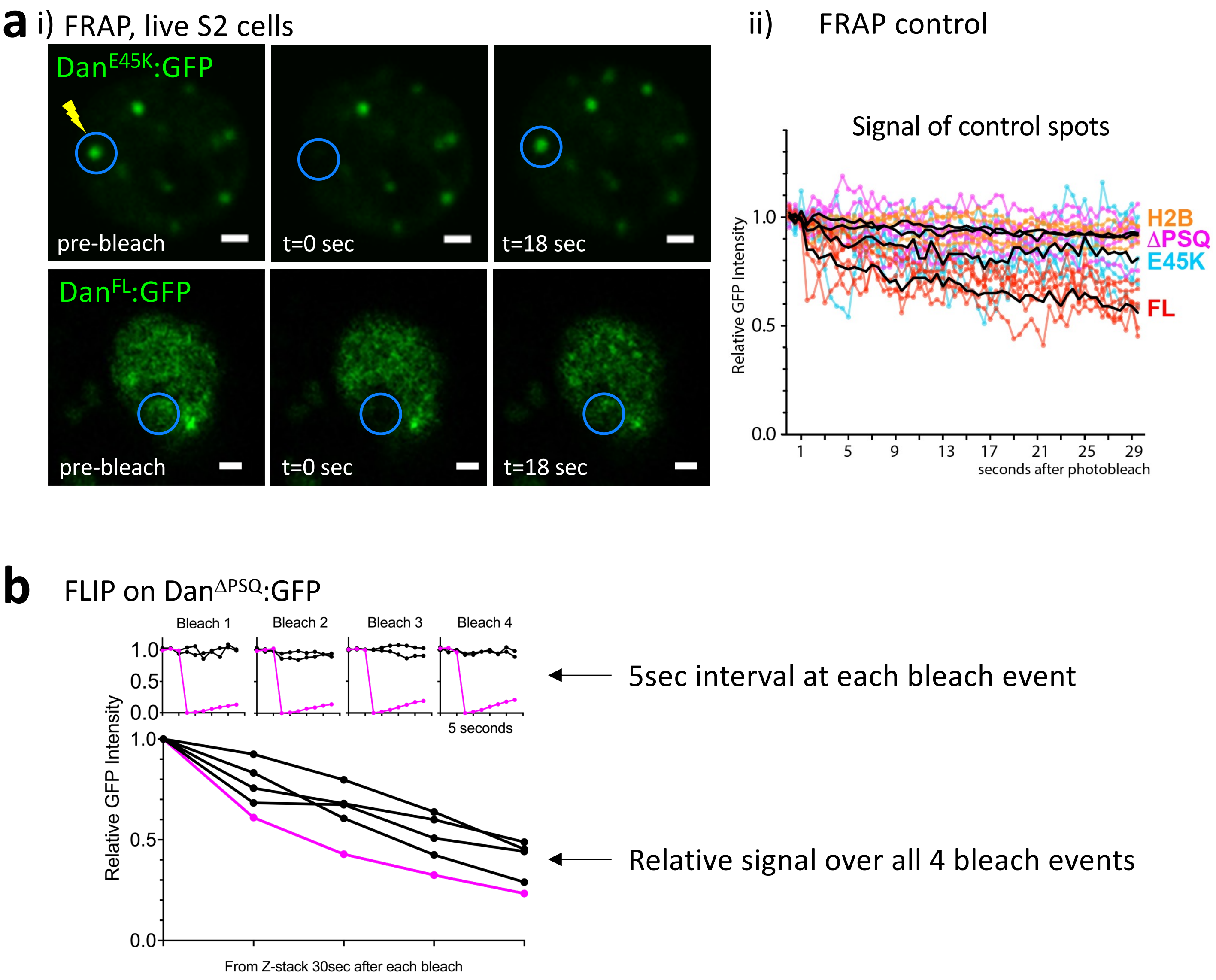
a
i) FRAP, live S2 cells
Dan^{E45K}:GFP
pre-bleach
t=0 sec
t=18 sec
Dan^{FL}:GFP
pre-bleach
t=0 sec
t=18 sec
ii) FRAP control
Signal of control spots
Relative GFP Intensity
1.0
0.5
0.0
1 5 9 13 17 21 25 29
seconds after photobleach
H2B
ΔPSQ
E45K
FL
b
FLIP on Dan$^{\Delta PSQ}$:GFP
Bleach 1
Bleach 2
Bleach 3
Bleach 4
1.0
0.5
0.0
5 seconds
Relative GFP Intensity
From Z-stack 30sec after each bleach
5sec interval at each bleach event
Relative signal over all 4 bleach events

Supplemental Figure 4 (Supplement to Figure 3)

ai, Representative Fluorescence Recovery After Photobleaching (FRAP) images of Dan^{E45K}:GFP and Dan^{FL}:GFP. Scale bars, 1μm. **ii,** Quantification of control (no bleach) condensates or regions for each condition shown in Fig 3a; Dan^{FL}:GFP (n=6 condensates), $Dan^{\Delta PSQ}$:GFP (n=6), Dan^{E45K}:GFP (n=5), and H2B:GFP control (n=4). Black line shows the mean of the traces for each. Source data are provided as a Source Data file.

b, Quantification of fluorescent intensity of condensates from an example single, representative $Dan^{\Delta PSQ}$:GFP cell after four successive bleaching events (fluorescence loss in photobleaching, FLIP). Pink trace represents the bleached condensate, and black traces represent non-bleached condensates in the same cell. Four small traces at the top show GFP intensity during a five second window relative to the GFP intensity at the onset of each photobleach (two black traces are nearest neighboring condensates, indicating the specificity of the bleached condensate). Bottom trace shows GFP intensity 30 seconds after each of the four bleach events relative to the original GFP intensity at the beginning of the experiment. Source data are provided as a Source Data file.

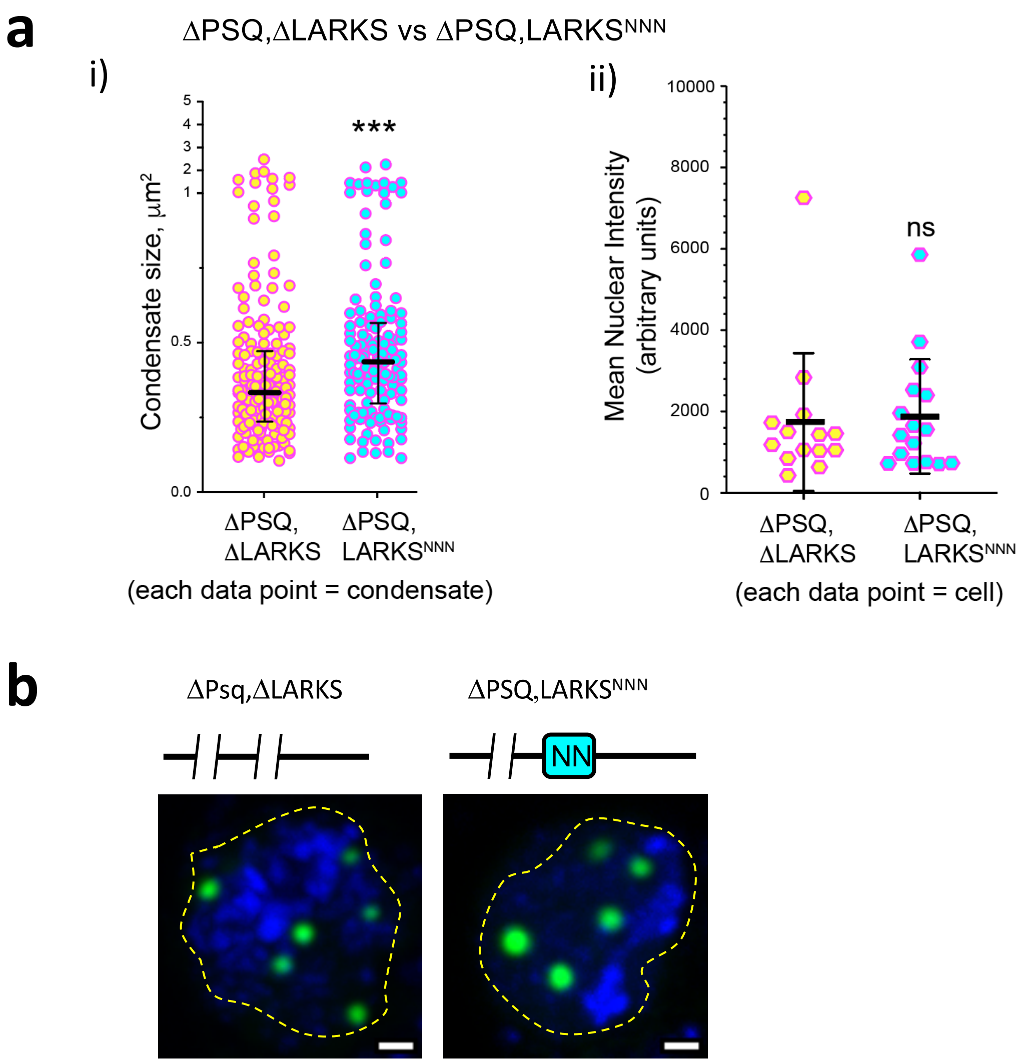
a
ΔPSQ,ΔLARKS vs ΔPSQ,LARKSNNN
i)
Condensate size, μm2
5
4
3
2
1
0.5
0.0
***
ΔPSQ, ΔLARKS
ΔPSQ, LARKSNNN
(each data point = condensate)
ii)
Mean Nuclear Intensity (arbitrary units)
10000
8000
6000
4000
2000
0
ns
ΔPSQ, ΔLARKS
ΔPSQ, LARKSNNN
(each data point = cell)
b
ΔPsq,ΔLARKS
ΔPSQ,LARKSNNN
NN

Supplemental Figure 5 (Supplement to Figure 5)

a, Graph shows quantification of Dan$^{\Delta PSQ\Delta LARKS}$:GFP and Dan$^{\Delta PSQ,NNN}$:GFP condensate sizes among cells expressing comparable levels of GFP. **i,** GFP Mean nuclear intensity (IU) per cell. Each point is a single cell. n(Dan$^{\Delta PSQ\Delta LARKS}$)=14 cells, n(Dan$^{\Delta PSQ,NNN}$)=16 cells; **ii,** Condensate size. Each point is a single hub. ∆PSQ,∆LARKS n=138, ∆PSQ,NNN n=171. Error bars show mean ± SD; two-tailed Mann Whitney, ****p*=0.0003, ns *p*=0.6971.

b, Representative 3D projection image of S2 cells. Schematic of construct is shown above each image. The dotted yellow line denotes the nuclear border. Scale bar, 1μm. Source data are provided as a Source Data file.

Supplementary Table 1
Primers used to generate *hb* FISH probe

Forward Primers	Reverse Primers
GAGGCTGCCGCTTGATTAACAC	ATCCAAGCAGGATCTCAGTAGC
AGGCTACTGAGATCCTGCTTGG	GCGTGGTTTGCTGTGGGAAATG
AAACGTGCCCTCCTGTTAAGTG	TAGATGGGCGGATATGGGTCTC
TCGAGTTCATCCCTCAACCTCC	GACAACAATTCCACTCAGCCAC
TGGCTGAGTGGAATTGTTGTCG	TCCTCCTTTCCCAGCTATTGAC
AGTCAATAGCTGGGAAAGGAGG	AGATGTGAGCCCAGTGTAATCC
GGGCAACTTTAAGCCCAGACAC	GGCATGGACTTTCGGCAATTAG
TCCAACATTACGCAGTACGCAG	CACTATTGTTTGGCCGCATAGC
