# Supplementary figures and images for: Dan forms condensates in neuroblasts and regulates nuclear architecture and progenitor competence in vivo
Source: Nat Commun. 2024 Jun 14;15:5097. doi: 10.1038/s41467-024-49326-6 (PMC11178893; doi:10.1038/s41467-024-49326-6)

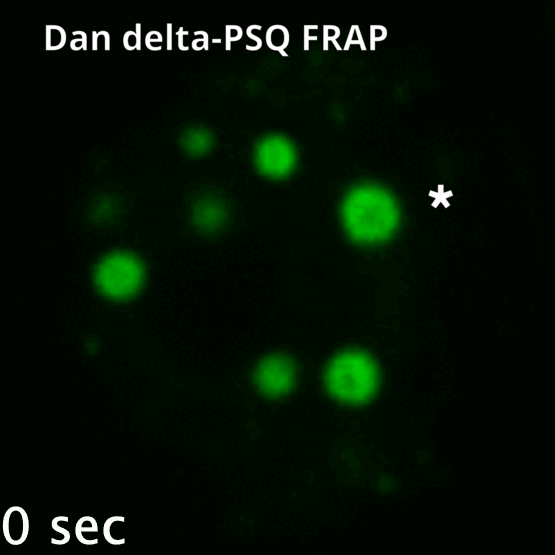

Supplement: Supplementary file 3 — Supplementary Movie 1 [file 41467_2024_49326_MOESM3_ESM.gif]

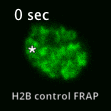

Supplement: Supplementary file 4 — Supplementary Movie 2 [file 41467_2024_49326_MOESM4_ESM.gif]

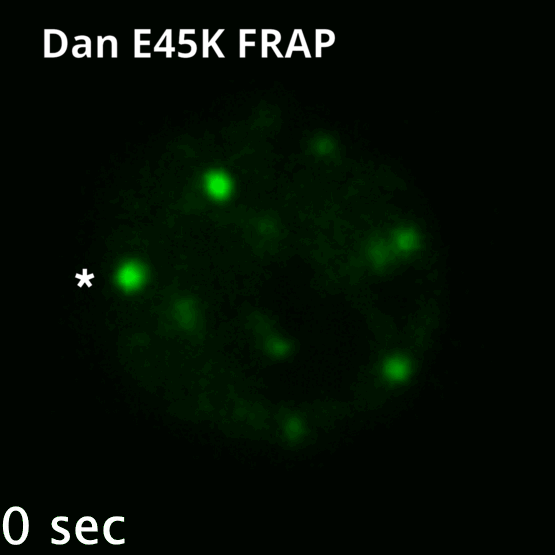

Supplement: Supplementary file 5 — Supplementary Movie 3 [file 41467_2024_49326_MOESM5_ESM.gif]

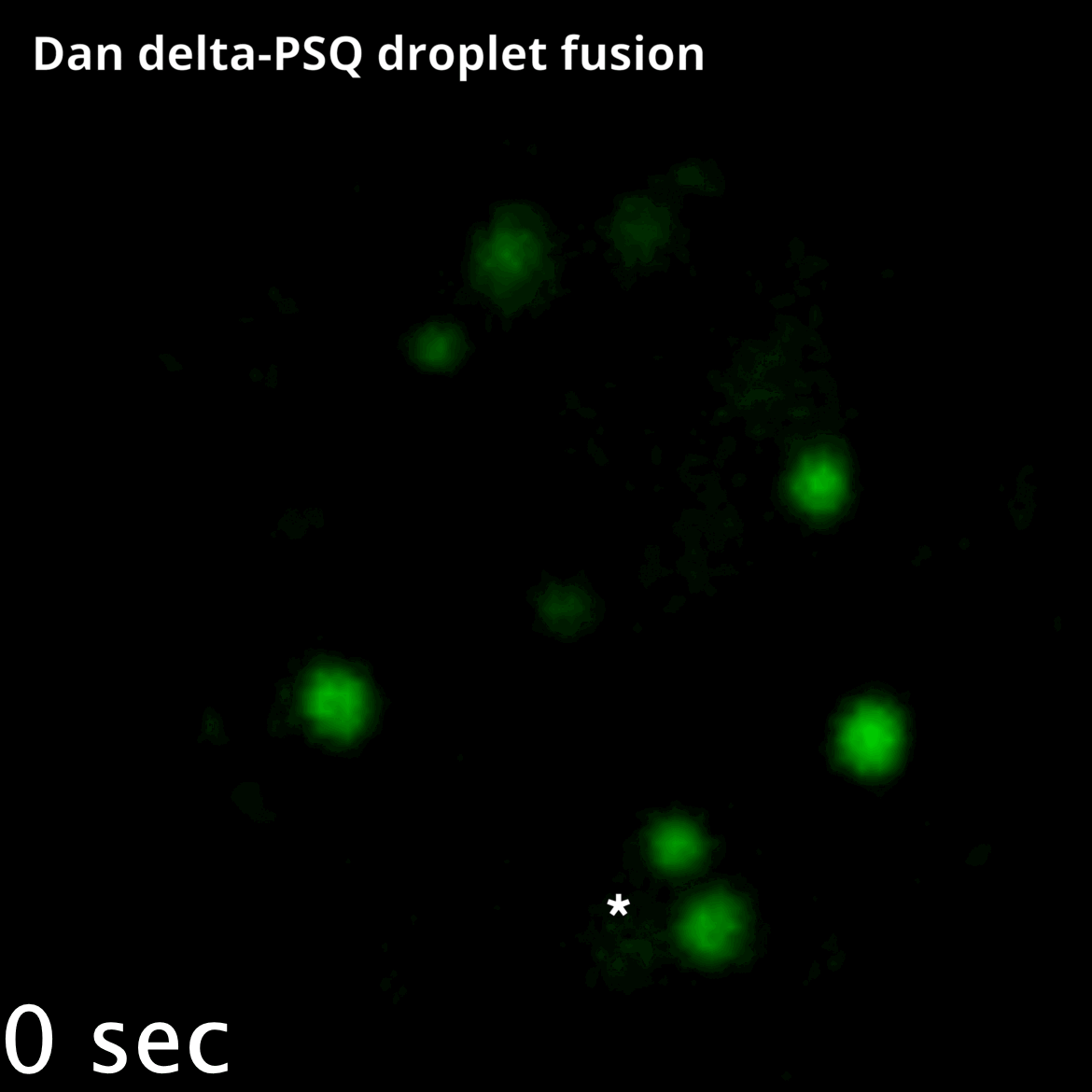

Supplement: Supplementary file 6 — Supplementary Movie 4 [file 41467_2024_49326_MOESM6_ESM.gif]
