# Supplementary material for: Dan forms condensates in neuroblasts and regulates nuclear architecture and progenitor competence in vivo
Source: Nat Commun. 2024 Jun 14;15:5097. doi: 10.1038/s41467-024-49326-6 (PMC11178893; doi:10.1038/s41467-024-49326-6)
Supplement: Supplementary file 7 — Reporting Summary [file 41467_2024_49326_MOESM7_ESM.pdf]

nature portfolio

Corresponding author(s): Minoree Kohwi

Last updated by author(s): May 22, 2024

# Reporting Summary

Nature Portfolio wishes to improve the reproducibility of the work that we publish. This form provides structure for consistency and transparency in reporting. For further information on Nature Portfolio policies, see our Editorial Policies and the Editorial Policy Checklist.

## Statistics

For all statistical analyses, confirm that the following items are present in the figure legend, table legend, main text, or Methods section.

| n/a | Confirmed | |
|---|---|---|
| ☐ | ☒ | The exact sample size (*n*) for each experimental group/condition, given as a discrete number and unit of measurement |
| ☐ | ☒ | A statement on whether measurements were taken from distinct samples or whether the same sample was measured repeatedly |
| ☐ | ☒ | The statistical test(s) used AND whether they are one- or two-sided<br>*Only common tests should be described solely by name; describe more complex techniques in the Methods section.* |
| ☒ | ☐ | A description of all covariates tested |
| ☐ | ☒ | A description of any assumptions or corrections, such as tests of normality and adjustment for multiple comparisons |
| ☐ | ☒ | A full description of the statistical parameters including central tendency (e.g. means) or other basic estimates (e.g. regression coefficient) AND variation (e.g. standard deviation) or associated estimates of uncertainty (e.g. confidence intervals) |
| ☒ | ☐ | For null hypothesis testing, the test statistic (e.g. *F*, *t*, *r*) with confidence intervals, effect sizes, degrees of freedom and *P* value noted<br>*Give P values as exact values whenever suitable.* |
| ☒ | ☐ | For Bayesian analysis, information on the choice of priors and Markov chain Monte Carlo settings |
| ☒ | ☐ | For hierarchical and complex designs, identification of the appropriate level for tests and full reporting of outcomes |
| ☒ | ☐ | Estimates of effect sizes (e.g. Cohen's *d*, Pearson's *r*), indicating how they were calculated |

*Our web collection on statistics for biologists contains articles on many of the points above.*

## Software and code

Policy information about availability of computer code

| | |
|---|---|
| Data collection | Zeiss 700 confocal image data were collected using the Zen software, and Nikon spinning disk image data were collected using NIS-Elements software. |
| Data analysis | ImageJ/FIJI was used for all image processing and analysis, PRISM was used for statistical analysis, PANTHER 17.0 was used for GO analysis, MaxQuant environment v.1.6.1.0 used for LC-MS/MS data analysis, IUPred3 was used for intrinsic disorder prediction. |

For manuscripts utilizing custom algorithms or software that are central to the research but not yet described in published literature, software must be made available to editors and reviewers. We strongly encourage code deposition in a community repository (e.g. GitHub). See the Nature Portfolio guidelines for submitting code & software for further information.

## Data

Policy information about availability of data

All manuscripts must include a data availability statement. This statement should provide the following information, where applicable:

- Accession codes, unique identifiers, or web links for publicly available datasets
- A description of any restrictions on data availability
- For clinical datasets or third party data, please ensure that the statement adheres to our policy

The data generated during the current study are available in the Source Data File and are also available from the corresponding author upon reasonable request.

## Human research participants

Policy information about studies involving human research participants and Sex and Gender in Research.

| | |
|---|---|
| Reporting on sex and gender | This study contains no human research participants. |
| Population characteristics | This study contains no human research participants. |
| Recruitment | This study contains no human research participants. |
| Ethics oversight | This study contains no human research participants. |

Note that full information on the approval of the study protocol must also be provided in the manuscript.

# Field-specific reporting

Please select the one below that is the best fit for your research. If you are not sure, read the appropriate sections before making your selection.

☒ Life sciences ☐ Behavioural & social sciences ☐ Ecological, evolutionary & environmental sciences

For a reference copy of the document with all sections, see nature.com/documents/nr-reporting-summary-flat.pdf

# Life sciences study design

All studies must disclose on these points even when the disclosure is negative.

| | |
|---|---|
| Sample size | All experiments were performed following field standards described in numerous publications whose results have been validated by multiple groups. These are all referenced in the manuscript. At least three independent animals that were carefully matched by genotype and by developmental stage were included for both the control and experiment. Competence experiments include analyzing 10-15 neuroblast lineages per embryo and at least three embryos per genotype and stage. Controls were performed in parallel and confirmed to match results of several previous publications, indicating high reproducibility and consistency in results and methodology. |
| Data exclusions | No data was excluded. |
| Replication | For embryo competence experiments, Dan misexpression using UAS-mediated constructs showed similar results between Dan without epitope tags and Dan with epitope tags (myc, GFP), as well as UAS-Dan inserted into different chromosomal locations, indicating reproducibility of observations both within this study and compared to previously published results. For DNA FISH experiments, YW (wild type) embryo analyses were included, to validate reproducibility and consistency with previously published results. |
| Randomization | N/A to this study. All samples were allocated based on genotype or construct and were compared to associated controls. |
| Blinding | For S2 cell droplet size analysis, the researcher was blind to which cells belonged to which group. For embryo-based experiments, immunostaining made it not possible to be blind to the genotype, but results were verified through two independent quantifications. |

# Reporting for specific materials, systems and methods

We require information from authors about some types of materials, experimental systems and methods used in many studies. Here, indicate whether each material, system or method listed is relevant to your study. If you are not sure if a list item applies to your research, read the appropriate section before selecting a response.

## Materials & experimental systems

| n/a | Involved in the study | |
|---|---|---|
| ☐ | ☒ | Antibodies |
| ☐ | ☒ | Eukaryotic cell lines |
| ☒ | ☐ | Palaeontology and archaeology |
| ☐ | ☒ | Animals and other organisms |
| ☒ | ☐ | Clinical data |
| ☒ | ☐ | Dual use research of concern |

## Methods

| n/a | Involved in the study | |
|---|---|---|
| ☒ | ☐ | ChIP-seq |
| ☒ | ☐ | Flow cytometry |
| ☒ | ☐ | MRI-based neuroimaging |

## Antibodies

| | |
|---|---|
| Antibodies used | Primary antibodies: anti-Eve (1:50, Mouse, #3C10 Developmental Studies Hybridoma Bank), anti-HA (1:500, Rat, #11867423001 |

| | |
|---|---|
| | Sigma Roche), anti-Dan (1:1000, Rabbit, (Kohwi et al., 2013)), anti-Myc (1:100, Rabbit, #ab9106 Abcam), anti-MSL2 (1:300, Rabbit, kind gift from Dr. Mitzi Kuroda, Harvard University), anti-Dpn (1:100, Rat, #ab195172 Abcam), anti-Wor (Rat, #ab196362 Abcam), anti-Lamin (Rabbit, R-836, kind gift from Dr. Paul Fischer, Stonybrook).<br>Secondary antibodies: Goat anti-Rabbit IgG, Alexa Fluor 555 (#A21429 Invitrogen), Donkey anti-Rat IgG, Biotin-SP (#712065153 Jackson ImmunoResearch), Donkey anti-Mouse IgG, Alexa Fluor 647 (#715605151 Jackson ImmunoResearch), Goat anti-Mouse IgG, DyLight 550 (#A90516D3 Bethyl Laboratories), Goat anti-Rabbit IgG, Alexa Fluor 488 (#A11034 Invitrogen), Donkey anti-Rat IgG, DyLight 550 (#A110337D3 Bethyl Laboratories). All Invitrogen secondaries were used at 1:400, Bethyl lab secondaries at 1:100. Streptavidin Cy3 (1:500, #SA1010 Invitrogen). DAPI (200ng/ml, #D3571 Invitrogen). |
| Validation | All commercial antibodies have been validated by the manufacturer, citations for non-commercial antibodies are supplied in the manuscript |

## Eukaryotic cell lines

Policy information about cell lines and Sex and Gender in Research

| | |
|---|---|
| Cell line source(s) | Drosophila S2 cells sourced from ThermoFisher Scientific (#R69007) |
| Authentication | Cell line was not authenticated by the lab. It was purchased commercially from ThermoFisher. |
| Mycoplasma contamination | Cell lines were tested for contamination of Mycoplasma as part of ThermoFisher's quality testing. |
| Commonly misidentified lines<br>(See ICLAC register) | Commonly misidentified lines were not used |

## Animals and other research organisms

Policy information about studies involving animals; ARRIVE guidelines recommended for reporting animal research, and Sex and Gender in Research

| | |
|---|---|
| Laboratory animals | Drosophila melanogaster, embryo - adult ages |
| Wild animals | The study did not involved wild animals |
| Reporting on sex | Yellow white Drosophila melaongaster flies were used in this study. Neuroblast competence experiments were performed on male embryos due to genotypic constraints (i.e. only male embryos lack the gal80 transgene, allowing transgene overexpression). However, sex is not a determining factor in this experiment, as competence results in wild type/control animals fully reproduced results from prior publications in which male and female embryos were mixed in the analyses (Kohwi et al., 2013). |
| Field-collected samples | The study did not involve field-collected samples |
| Ethics oversight | No ethical oversight was required as only D. melanogaster animals and cell lines were used |

Note that full information on the approval of the study protocol must also be provided in the manuscript.
